# Three New Phytoecdysteroids Containing a Furan Ring from the Roots of *Achyranthes bidentata* Bl

**DOI:** 10.3390/molecules16075989

**Published:** 2011-07-18

**Authors:** Qiu-Hong Wang, Liu Yang, Hai Jiang, Zhi-Bin Wang, Bing-You Yang, Hai-Xue Kuang

**Affiliations:** Key Laboratory of Chinese Materia Medica, Heilongjiang University of Chinese Medicine), Ministry of Education, No. 24 HePing Road, XiangFang District, Harbin 150040, China

**Keywords:** *Achyranthes bidentata* Bl., phytoecdysteroid, niuxixinsterone

## Abstract

Three new phytoecdysteroid compounds, named niuxixinsterone A (**1**), B (**2**) and C (**3**) with acetal functions in the side-chain were isolated from *Achyranthes bidentata* Bl. The structures were established as (20*R*,22*R*,24*S*)-20-*O*,22-*O*-(5′-hydroxymethyl)-furfurylidene-2β,3β,14α,25-tetrahydroxy-5β-ergost-7-en-6-one (**1**), (20*R*,22*R*)-20-*O*,22-*O*-(5′-hydroxymethyl)-furfurylidene-2β,3β,25-trihydroxy-14β-methyl-18-nor-5β-cholesta-7,12-dien-6-one (**2**) and (20*R*,22*R*,25*R*)-20-*O*,22-*O*-(5′-hydroxymethyl)-furfurylidene-2β, 3β,5β,14α,26-pentahydroxycholest-7-en-6-one (**3**) by means of spectroscopic evidence.

## 1. Introduction

*Achyranthes bidentata* Bl., a member of *Amaranthaceae*, is an erect perennial herbaceous plant widely distributed and grown in hilly districts of India, China, Japan and Java. The roots of *A. bidentata* named “Niuxi” in Chinese, is an important medicinal herbal and documented in the Chinese Pharmacopeia. It is usually prescribed by practitioners of Traditional Chinese Medicine (TCM) as a tonic, emmenagogue, antiarthritic, diuretic, and antifertility agent to nourish the liver and kidneys, strengthen bones and muscles, and invigorate circulation [1]. Modern pharmacological studies have shown that the *A. bidentata* possesses immunostimulant [2,3], uteri-excitant and antifertility [4,5], antitumor [6], analgestic, antibacterial, anti-inﬂammatory [7], cognition-enhancing [8], antisenile [9], and anti-osteoporosis [10] activities. Its main constituents include polysaccharides [2,3], saponins [11,12] and ecdysteroids [13,14].

**Figure 1:**
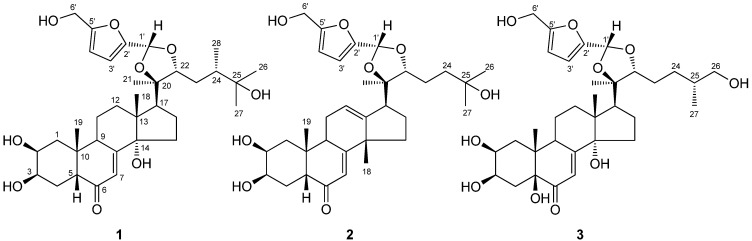
Structures of compounds **1**-**3**.

In this paper, we describe the isolation and structure elucidation of three new phytoecdysteroids with acetal functions [15] (Figure 1) isolated from the EtOH extracts of *A. bidentata*. In previous experiments, we discovered ecdysterone, inokosterone and serfurosterone A [16]. The three new phytoecdysteroids had a similar structure to serfurosterone A, and we presumed they might have similar pharmacological activity. The structure determination of the phytoecdysteroids from *A. bidentata* could establish a basis for further pharmacological experiments.

Here, we describe the isolation and structure elucidation of three new phytoecdysteroid compounds on the basis of the spectroscopic analysis, including 1D, 2D-NMR techniques and HRESIMS.

## 2. Results and Discussion

Compound **1** was obtained as a white amorphous powder and had a [M+Na]^+^ ion peak at *m/z* 625.3359 in the HRESIMS, corresponding to a molecular formula of C_34_H_50_O_9_. The UV spectrum was consistent with presence of a 7-en-6-one chromophore in an ecdysteroid, with a maximum value at 248 nm. By analyzing the ^1^H- and ^13^C-NMR spectroscopic data, it was determined that **1** was an ecdysone analog with a furan ring-containing substituent.

In the ^1^H-NMR spectrum of **1**, five methyl singlets at *δ*_H_ 1.00 (×2), 1.53, 1.28 and 1.36 were attributed to CH_3_-18, CH_3_-19, CH_3_-21, CH_3_-26 and CH_3_-27, respectively. The CH_3_-28 signal appeared as a doublet at *δ*_H_ 1.19 (*J* = 6.8 Hz). In addition, signals for three olefin protons (H-7, 3′ and 4′), two hydroxymethyl protons, and an acetal proton were observed in the spectrum.

**Figure 2:**
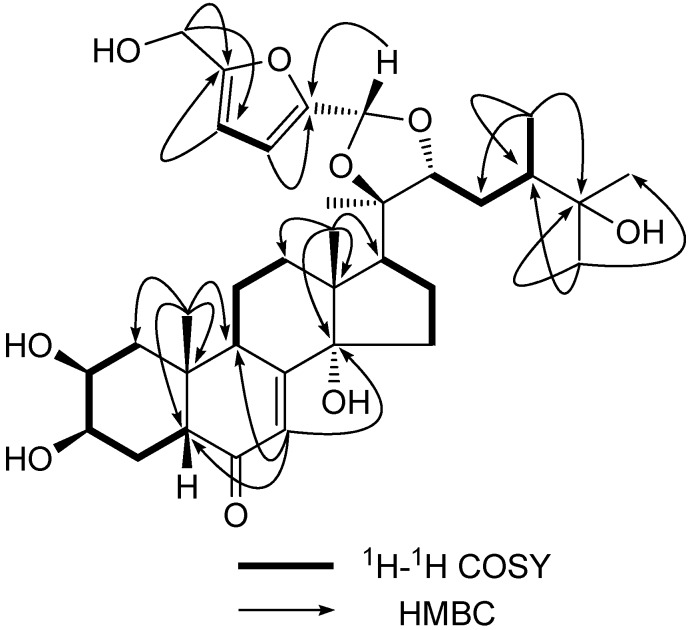
Selected 2D NMR correlations of Niuxixinsterone A(**1**).

The ^13^C-NMR spectrum of **1** had one carbonyl at *δ*_C_ 203.4 (C-6), three oxymethine at *δ*_C_ 68.2, 68.1 and 85.4 (C-2, 3 and 22), three oxyquaternary carbons at *δ*_C_ 80.4, 85.5 and 72.1 (C-14, 20 and 25), six olefinic carbons at *δ*_C_ 121.7, 165.4, 152.2, 110.1, 107.8 and 157.4 (C-7, 8, 2′, 3′, 4′ and 5′), together with an acetal carbon at *δ*_C_ 97.8. The full connectivity of **1** was deduced from the HSQC, ^1^H-^1^H-COSY and HMBC correlations (Figure 2). 

The stereochemistry of **1** was elucidated from NOESY data and ^1^H-^1^H coupling constants. The H_3_-19/H_β_-11, H_3_-19/H_β_-1, and H_3_-19/H_β_-5 correlations in the NOESY spectrum were indicative of a *cis*-A/B ring junction, whereas the H_β_-11/H_3_-18, H_β_-15/H_3_-18, H_β_-12/H_3_-18, and H-9/H_α_-12 cross-peaks indicated a *trans*-C/D ring junction. The relative configurations of the hydroxyl groups at C-2 and C-3 were elucidated from the coupling constant (11.8 Hz, brd) between H_β_-1 and H-2, a broad singlet of H-3, HMBC correlations between H_3_-19 and C-1, and NOESY correlation between H-2 and H_α_-4. A NOESY correlation between H_α_-12 and H-17 indicated that C-17 side-chain was β-oriented [17]. All of the above data were in good agreement with the structural features of 2β,3β,14α-trihydroxy-5β-cholest-7-en-6-one.

**Figure 3:**
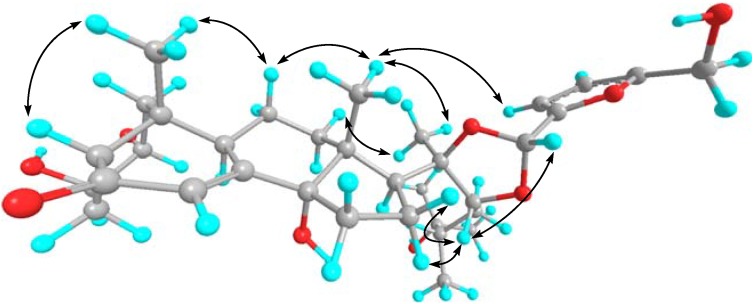
Selected NOESY correlations of niuxixinsterone A(**1**).

In compound **1** the high chemical shifts of C-20 (*δ*_C_ 85.5) and C-22 (*δ*_C_ 85.4) proved the oxygen substitution. The NOESY correlation between H-22 and H-1′ in **1** and the chemical shift of C-1′ (97.8 ppm) verified the existence of an acetal-type five-membered ring. Moreover, the H-1′/C-2′, H-3′/C-2′, H-4′/C-5′, H-6′/C-4′ and H-6′/C-5′ HMBC cross-peaks revealed a 5-hydroxymethyl-furfurylidene substituent on C-1′. The characteristic ^13^C-NMR chemical shifts and the low coupling constant value ^3^*J*_H-30, 31_ = 3.2 Hz furnished further support for the structure [16]. Furthermore, the H_β_-12/H_3_-21, H_3_-18/H_3_-21, H_3_-18/H-3′, H-22/H_2_-16 and H-22/H-1′ NOESY correlations revealed the absolute configuations of 20*R* and 22*R* as shown in Figure 3. In addition, the absolute configurations of C-20 and C-22 were consistent with previously reported similar phytoecdysteroids with acetal functions [16]. Comparison of ^13^C-NMR chemical shifts of C-23~28 in **1** with 24-*epi* makisterone A and makisterone A [18,19] indicated a 24*S* configuration. Accordingly, compound **1** was identified as (20*R*,22*R*,24*S*)-20-*O*,22-*O*-(5′-hydroxymethyl)-furfurylidene-2β,3β,14α,25-tetrahydroxy-5β-ergost-7-en-6-one.

Compound **2**, a white amorphous powder, was assigned a molecular formula of C_33_H_46_O_8_ by HRESIMS, which exhibited a [M+Na]^+^ ion peak at *m/z* 593.3091. The UV spectrum was consistent with presence of the ecdysteroid chromophore as above. Extensive NMR analysis of the parent nucleus indicated that compounds **2** and **1** were structurally different in terms of the presence of additional one olefin signal and the loss of one oxyquaternary carbon signal.

Comparison of ^1^H- and ^13^C-NMR data from the spectra of **2** and (20*R*,22*R*)-2β,3β,20,22,26-pentahydroxy-14β-methyl-18-nor-5β-cholesta-7,12-dien-6-one [13] revealed the close similarity between them, with practically identical values for C-1 to C-19, which suggests the structure of the tetracyclic ring system of these two ecdysteroids to be the same. The attachment of the parent nucleus was also determined by ^1^H-^1^H COSY and HMBC spectrum. The H_3_-18/C-8, 13 and 14, H_α_-17/C-12, 13 and 14, and H_α_-12/C-9, 11, 13, 14 HMBC cross-peaks and the H_α_-11/H_α_-12 ^1^H-^1^H COSY correlations revealed that the linkage position of double bond into position C-12 and C-13 and CH_3_-18 substituent on C-14. The 14β-CH_3 _configuration was confirmed in ^1^H-^1^H-ROESY spectrum by observation of NOE contacts of H-7/H_β_-15 and H-9/H_α_-15.

Detailed comparison of chemical shifts of C-17 side-chain of compound **2** with **1** indicated that both compounds had the same side-chain, but compound **2** had one less methyl signal at C-24. Thus, the structure of **2** was formulated as (20*R*,22*R*)-20-*O*,22-*O*-(5′-hydroxymethyl)-furfurylidene-2β,3β,25-trihydroxy-14β-methyl-18-nor-5β-cholesta-7,12-dien-6-one.

Compound **3**, a white amorphous powder, was established by HRESIMS (m/z 627.3150 [M+Na]^+^) as C_33_H_48_O_10_ with a maximal UV absorption at 247 nm. Comparison of the ^13^C-NMR spectroscopic data of the tetracyclic ring system between **3** and **1** indicated that the major difference was a methine at *δ*_C_ 51.4 (C-5) in **1** being replaced by an oxyquaternary at *δ*_C_ 80.0 in **3**. The HMBC correlations between H-7 and H_3_-19 with the signal at *δ*_C_ 80.0, respectively, which established the presence of an OH substituent at C-5. The 5β-OH configuration was indicated by the upfield resonance for the CH_3_-19 at *δ*_C_ 17.1 [20].

In compound **3**, three methyl singlets at *δ*_H_ 1.12, 0.99 and 1.38 were reasonably attributed to the methyl groups at C-18, C-19 and C-21 by a HMBC experiment. Additionally, the methyl doublet *δ*_H_ 1.04 is correlated in HMBC to C-23, C-24 and C-26, which is only in agreement with the presence of this methyl group at C-25. Comparison of ^13^C-NMR chemical shifts of C-23～27 in **3** with palythoalone B [17] and 25*R*-inokosterone [21] indicated a 25*R* configuration.

Furthermore, the ^1^H-^1^H COSY and HMBC correlations implied that **3**also displayed a furan ring-containing substituent at C-20 and C-22. The absolute configurations of substituents at positions 20 and 22 were derived from the observed NOE contacts and comparison with the known compound [16]. Consequently, **3** was elucidated as (20*R*,22*R*,25*R*)-20-*O*,22-*O*-(5′-hydroxymethyl)-furfurylidene-2β,3β,5β,14α,26-pentahydroxycholest-7-en-6-one.

Many plants have been found to be rich in ecdysteroids [22]. A multiplicity of ecdysteroids have been isolated from members of the *Amaranthaceae* [23,24,25,26,27]. The common structural features are characteristic of the ecdysteroids: ∆7-6-keto grouping in ring B; *cis*-junction of A/B rings; hydroxyl groups in positions 1, 2, 3, 5, 11, and 14 of the steroidal core; and the side chain usually containing an (*R*)-C22-group. In most cases, phytoecdysteroids are isolated in a free state, although many their derivatives (ethers, esters, and glycosides) have also been found.

However, the distribution of ecdysteroids with a furan ring in the side-chain in natural sources is very limited. They have been found so far only in the plant *Serratula wolffii* [16]. The new isolates **1**-**3** constitute a series of ecdysteroids containing a furan ring in the side-chain, all of them with an acetal group at C-1′, which have not been reported previously from *A. bidentata*. It is difficult to decide whether the three ecdysteroids are originally present in the plant or if it is artifacts formed during root drying, but we consider that these compounds are genuine compounds as they can be characterized by their UPLC-MS retention time and fragmentation pattern in ethanol extracts of fresh root. It is well know that 20,22-condensation reaction can take place under certain conditions [28], so compounds **1**-**3** are new endogenous compounds.

## 3. Experimental

### 3.1. General

The melting points (uncorrected) were measured on a Kofler micromelting point apparatus. Optical rotations were measured with a PE-241 digital polarimeter. IR spectra were recorded on an IR-47 spectrometer. The NMR spectra were recorded on Bruker DPX 400 (400 MHz for ^1^H-NMR and 100 MHz for ^13^C-NMR), respectively. Chemical shifts are given as *δ* values with reference to tetramethylsilane (TMS) as an internal standard, and coupling constants are given in Hz. The HRESIMS analyses were conducted on IonSpec Ultima 7.0T FTICR. Preparative HPLC (Waters, Delta 600-2487) was performed on Pegasil ODS II (5 μm, 10 × 250 mm, Senshu Pak, Japan). Macroporous absorption resin (D101 Crosslinked Polystyrene, Nan Kai, Tian Jin, China) was employed for column chromatography. Silica gel (100–200 mesh) for column chromatography and silica gel H for TLC were obtained from Qingdao Marine Chemical Factory, Qingdao, Shandong Province, China. ODS-A (120 A, 50 μm) was obtained from YMC Co. 

### 3.2. Plant Material

The roots of *A. bidentata* were purchased from the Medicinal Materials Planting Base of Anhui University of Traditional Chinese Medicine in 2007. The original plant was identified by Zhenyue Wang of Heilongjiang University of Chinese Medicine. A voucher specimen (No. 20071062) was deposited at the Herbarium of Heilongjiang University of Chinese Medicine, China.

### 3.3. Extraction and Isolation

The air dried roots of *A. bidentata* (12 kg) were ground to the particle size passing through standard No.10 mesh sieve and extracted with 95% EtOH (3 × 10 L) for 2 h. The EtOH extracts (5.5 kg) were concentrated under reduced pressure and fractioned by D101 macroporous resin column (8 × 60 cm) with H_2_O, 50% and 95% EtOH-H_2_O to give three fractions (H_2_O fraction, 50% EtOH-H_2_O fraction, 95% EtOH-H_2_O fraction). The 50% EtOH-H_2_O fraction, which showed potent proliferating activities on osteoblast-like cell formation, was subjected to further isolation. Thus, the fraction (108 g) was column chromatographed on silica gel with a gradient of CH_2_Cl_2_/MeOH (30:1 to 3:1) solvents as eluents to afford eight fractions: Fr.4 (20 g; eluted with CH_2_Cl_2_/MeOH 20:1 to 5:1) was further submitted to silica gel chromatography, and afforded four subfractions A1–A4. Compounds **1** (15.1 mg, t_R_ = 27.8 min) and **2** (17.5 mg, t_R_ = 38.6 min) were obtained by prep. HPLC chromatography of the sub-fraction A2 (1.3 g; eluted with MeOH/H_2_O 9:20). A3 (4 g; eluted with MeOH/H_2_O 1:5 to 1:0) was separated on ODS-A column, to produce five sub-fractions (B1–B5). The sub-fraction B3 (0.9 g) was purified by prep. HPLC with MeOH/H_2_O (2:5) to afford **3** (25.2 mg, t_R_ = 30.9 min).

*(20R,22R,24S)-20-O,22-O-(5′-Hydroxymethyl)-furfurylidene-2β,3β,14α,25-tetrahydroxy-5β-ergost-7-en-6-one* (*niuxixinsterone A*, **1**): white amorphous powder, mp: 227–228 °C. [*α*]_D_^20^ +55 (c 0.05, MeOH). IR (KBr) cm^−1^: 3460, 1660, 1454, 1380, 1062. UV (MeOH) λ_max_ (log ε) nm: 226 (4.10), 248 (3.20), 323(1.15). HRESIMS (positive ion mode) *m/z*: 625.3359 [M+Na]^+^, (calc. for C_34_H_50_O_9_Na 625.3353). ^1^H-NMR and ^13^C-NMR data are shown in Table 1.

**Table 1:** ^1^H- and ^13^C-NMR data for compounds **1**-**3** in C_5_D_5_N.(*δ* in ppm, *J* in Hz).

No	1			2			3	
	*δ*_H_	*δ*_C_		*δ*_H_	*δ*_C_		*δ*_H_	*δ*_C_
1α	2.12 (1H, m)	37.9		2.05 (2H, m)	37.9		2.21 (1H, m)	34.8
1β	1.90 (1H, m)						2.09 (1H, m)	
2α	4.17 (1H, d, 11.8)	68.2		4.17 (1H, m)	68.0		4.23 (1H, m)	67.9
3α	4.23 (1H, br. s)	68.1		4.42 (1H, br. s)	68.3		4.18 (1H, m)	69.9
4α	1.70 (1H, m)	32.4		1.90 (1H, m)	32.3		1.98 (1H, dd, 14.4, 2.8)	36.0
4β	1.97 (1H, m)			2.25 (1H, m)			2.10 (1H, m)	
5β	2.98 (1H, dd, 13.2, 3.6)	51.4		2.97 (1H, dd, 13.2, 4.0)	50.4			80.0
6		203.4			202.4			200.9
7	6.24 (1H, d, 2.0)	121.7		6.16 (1H, d, 2.4)	123.1		6.20 (1H, d, 2.4)	120.0
8		165.4		-	146.5			166.1
9α	3.52 (1H, m)	34.7		2.90 (1H, m)	39.5		3.60 (1H, m)	38.2
10		38.6			40.3			44.7
11α	1.78 (1H, m)	21.0		2.19 (1H, m)	21.7		1.88(2H, m)	21.4
11β	1.61 (1H, m)			1.86 (1H, m)				
12α	2.39 (1H, m)	31.7		6.04 (1H, m)	122.1		1.96 (1H, m)	31.7
12β	1.86 (1H, m)						1.88 (1H, m)	
13		47.7			173.6			47.8
14		84.0			48.9			83.9
15α	2.15 (1H, m)	31.5		1.83 (1H, m)	38.8		2.55 (1H, m)	31.7
15β	1.86 (1H, m)			1.50 (1H, m)			2.00 (1H, m)	
16α	2.15 (2H, m)	22.5		1.78 (2H, m)	26.2		2.47 (1H, m)	23.0
16β							1.98 (1H, m)	
17α	2.89 (1H, t, 8.6)	50.6		3.11 (1H, t, 9.2)	49.5		2.82 (1H, t, 9.2)	49.9
18β	1.00 (3H, s)	17.3		1.09 (3H, s)	25.4		1.12 (3H, s)	17.3
19β	1.00 (3H, s)	24.4		0.96 (3H, s)	23.3		0.99 (3H, s)	17.1
20		85.5			84.9			85.8
21	1.53 (3H, s)	22.6		1.45 (3H, s)	21.5		1.38 (3H, s)	21.3
22	4.20 (1H, dd, 10.4, 3.6)	85.4		4.23 (1H, dd, 10.0, 1.6)	83.4		4.14 (1H, dd, 9.6, 3.2)	82.9
23	2.39 (1H, m)	31.3		2.03 (1H, m)	26.2		1.85 (1H, m)	27.4
	1.86 (1H, m)			2.16 (1H, m)			1.52 (1H, m)	
24	1.94 (1H, m)	44.5		2.17 (1H, m)	42.2		1.90 (1H, m)	31.6
				1.82 (1H, m)			1.55 (1H, m)	
25		72.1			69.2		1.82 (1H, m)	36.7
26	1.28 (3H, s)	25.4		1.41 (3H, s)	29.6		3.63 (2H, m)	66.8
27	1.36 (3H, s)	28.9		1.43 (3H, s)	30.5		1.04 (3H, d, 6.4)	17.1
28	1.19 (3H, d, 6.8)	16.6						
1′	6.11 (1H, s)	97.8		6.21 (1H, s)	96.8		6.28 (1H, s)	96.4
2′		152.2			151.8			153.4
3′	6.66 (1H, d, 3.2)	110.1		6.73 (1H, d, 3.2)	110.3		6.61 (1H, d, 3.2)	109.1
4′	6.44 (1H, d, 3.2)	107.8		6.45 (1H, d, 3.2)	107.8		6.43 (1H, d, 3.2)	107.7
5′		157.4			157.6			157.3
6′	4.83 (2H, s)	57.2		4.85(2H, s)	57.2		4.86 (2H, s)	57.2

*(20R,22R)-20-O,22-O-(5′-Hydroxymethyl)-furfurylidene-2β,3β,25-trihydroxy-14β-methyl-18-nor-5β-cholesta-7,12-dien-6-one* (*niuxixinsterone* *B*,** 2**): white amorphous powder, mp: 230–231 °C. [*α*]_D_^20^ +46 (c 0.025, MeOH). IR (KBr) cm^−1^: 3472, 1655, 1460, 1380, 1059. UV (MeOH) λ_max_ (logε) nm: 224 (4.05), 248 (3.23), 321 (1.09). HRESIMS (positive ion mode) *m/z*: 593.3091 [M+Na]^+^, (calc. for C_33_H_46_O_8_Na 593.3090). ^1^H-NMR and ^13^C-NMR data are shown in Table 1.

*(20R,22R,25R)-20-O,22-O-(5′-Hydroxymethyl)-furfurylidene-2β,3β,5β,14α,26-pentahydroxycholest-7-en-6-one* (*niuxixinsterone* *C*,**3**): white amorphous powder, mp: 235–236 °C. [*α*]_D_^20^ +52 (c 0.06, MeOH). IR (KBr) cm^−1^: 3480, 1656, 1456, 1380, 1062. UV (MeOH) λ_max_ (logε) nm: 225 (4.09), 247 (3.51), 320 (1.07). HRESIMS (positive ion mode) *m/z*: 627.3150 [M+Na]^+^, (calc. for C_33_H_48_O_10_Na 627.3145). ^1^H-NMR and ^13^C-NMR data are shown in Table 1.

## 4. Conclusions

In conclusion, three new phytoecdysteroids, (20*R*,22*R*,24*S*)-20-*O*,22-*O*-(5′-hydroxymethyl)-furfurylidene-2β,3β,14α,25-tetrahydroxy-5β-ergost-7-en-6-one (1), (20*R*,22*R*)-20-*O*,22-*O*-(5′-hydroxy-methyl)-furfurylidene-2β,3β,25-trihydroxy-14β-methyl-18-nor-5β-cholesta-7,12-dien-6-one (2) and (20*R*,22*R*,25*R*)-20-*O*,22-*O*-(5′-hydroxymethyl)-furfurylidene-2β,3β,5β,14α,26-pentahydroxy-cholest-7-en-6-one (3), were isolated from the EtOH extract of *Achyranthes bidentata* Bl. The discovery of compounds 1-3 is a further addition to the diverse phytoecdysteroids compounds.

## References

[1] Meng D.L., Li X. (2001). The research development of *Achyranthes bidentata* Bl. Chin. J. Med. Chem..

[2] Li C.C., Hu X.G., Zhang W.X., Xie L.W., Zhang H.Y., Dong L. (2003). Eosinophils apoptosis, fas mRNA and bcl-2 mRNA expressions in asthma model of young rat and effects of *Achyranthes bidentata* polysaccharides. Zhonghua Er Ke Za Zhi.

[3] Chen X.M., Xu Y.J., Tian G.Y. (2005). Physical-chemical properties and structure elucidation of abPS isolated from the root of *Achyranthes bidentata*. Yao Xue Xue Bao.

[4] Yuan Y.J., Cui Y., Yu Y., Yong Y.H. (2002). Different mechanisms mediate the exciting effect about *Achyranthes bidentata* on the spike activity of the uterine smooth muscle in virgin rats. Chin. J. Vet. Sci. Technol..

[5] Liu J.H., Liang S.W., Wang S.M. (2006). A study of antiprocreat effect of *Achyranthes bidentata* saponin suppository. J. Henan Univ. Chin. Med..

[6] Hu J., Qi Y.X., Li Q.X., Shan B.E. (2005). The research of extract of *Achyranthes bidentata* Blume anti-tumor activity. Chin. J. Microbiol. Immunol..

[7] Gao C.K., Gao J., Ma R.L., Xu X.X., Huang P., Ni S.D. (2003). Research on analgesic and anti-inflammatory and invigorate circulation effects of total saponins of *Achyranthes*. Anhui Med. Pharm. J..

[8] Ma A.L., Guo H. (1998). Effect of *Achyranthes bidentata* on memory and endurance. Zhong Yao Cai.

[9] Deng H.B., Cui D.P., Jiang J.M., Feng Y.C., Cai N.S., Li D.D. (2003). Inhibiting effects of *Achyranthes bidentata* polysaccharide and *Lycium barbarum* polysaccharide on nonenzyme glycation in D-galactose induced mouse aging model. Biomed. Environ. Sci..

[10] Gao C.K. (2001). Studies on the preventive and curative effects of *Achyranthes bidentata* on osteoporosis induced by retinoic acid in rats. Prim. J. Chin. Mater. Med..

[11] Wang X.J., Zhu L.Z. (1996). Studies on the saponin constituents of Niu Xi (*Achyrathes bidentata*). J. Fourth Mil. Med. Univ..

[12] Li J.X., Hareyama T., Tezuka Y., Zhang Y., Miyahara T., Kadota S. (2005). Five new oleanolic acid glycosides from *Achyranthes bidentata* with inhibitory activity on osteoclast formation. Planta Med..

[13] Li X., Zhao W.T., Meng D.L., Qiao A.M. (2007). A new phytosterone from the roots of *Achyranthes bidentata*. Fitoterapia.

[14] Meng D.L., Li X., Wang J.H., Li W. (2005). A new phytosterone from *Achyranthes bidentata* Bl. J. Asian Nat. Prod. Res..

[15] Ecdybase..

[16] Liktor-Busa E., Simon A., Tóth G., Báthori M. (2008). The first two ecdysteroids containing a furan ring from *Serratula wolffii*. Tetrahedron Lett..

[17] Shigemori H., Sato Y., Kagata T., Kobayashi J. (1999). Palythoalones A and B, new ecdysteroids from the marine zoanthid *Palythoa australiae*. J. Nat. Prod..

[18] Miller R.W., Clardy J., Kozlowski J., Mikolajczak K.L., Plattne R.D. (1985). Phytoecdysteroids of *Diploclisia glaucescens* seed. Planta Med..

[19] Sena Fiho J.G., Duringer J., Maia G.L.A., Tavares J.F., Xavier H.S., Sobral da Silva M., da-Cunha E.V.L., Barbosa-Filho J.M. (2008). Ecdysteroids from *Vitex* species: Distribution and compilation of their ^13^C-NMR spectral data. Chem. Biodivers..

[20] Jayasinghe L., Kumarihamy B.M.M., Arundathie B.G.S., Dissanayake L., Hara N., Fujimoto Y. (2003). A new ecdysteroid, 2-deoxy-5β,20-dihydroxyecdysone from the fruits of *Diploclisia glaucescens*. Steroids.

[21] Zhu T.T., Liang H., Zhao Y.Y., Wang B. (2004). Isolation and structure identification of C-25 epimers of inokosterone from *Achyranthes bidentata* Blume. Yao Xue Xue Bao.

[22] Baltaev U.A. (2000). Phytoecdysteroids: Structure, sources, and biosynthesis in plants. Russ. J. Bioorg. Chem..

[23] Ikan R., Ravid U., Trosset D., Shulman E. (1971). Ecdysterone: An insect moulting hormone from *Achyranthes aspera* (*Amaranthaceae*). Experientia.

[24] Hikino H., Hikino Y., Nomoto K., Takemoto T. (1968). Cyasterone, an insect metamorphosing substance from *Cyathula capitata*: structure. Tetrahedron.

[25] Kobayashi M., Takemoto T., Ogawa S., Nishimoto N. (1967). The moulting hormone activity of ecdysterone and inokosterone isolated from *Achyranthis radix*. J. Insect Physiol..

[26] Nishimoto N., Shiobara Y., Inoue S.S., Fujino M., Takemoto T., Yeoh C.L., Oliveira F.D., Akisue G., Akisue M.K., Hashimoto G. (1988). Three ecdysteroid glycosides from *Pfaffia iresinoides*. Phytochemistry.

[27] Sarker S.D., Girault J.P., Lafont R., Dinan L.N. (1996). Ecdysteroids from *Gomphrena haageana* (*Amaranthaceae*). Biol. Syst. Ecol..

[28] Suksamrarn A., Pattanaprateep P. (1995). Selective acetylation of 20-hydroxyecdysone partial synthesis of some minor ecdysteroids and analogues. Tetrahedron.

